# Binding Specificity of a Novel Cyclo/Maltodextrin-Binding Protein and Its Role in the Cyclodextrin ABC Importer System from Thermoanaerobacterales

**DOI:** 10.3390/molecules28166080

**Published:** 2023-08-16

**Authors:** Jorge Aranda-Caraballo, Roberto A. Saenz, Alonso A. López-Zavala, Beatriz Velazquez-Cruz, Laura Espinosa-Barrera, Yair Cárdenas-Conejo, Andrés Zárate-Romero, Oscar Linares-Vergara, Juan A. Osuna-Castro, Edgar Bonales-Alatorre, Sara Centeno-Leija, Hugo Serrano-Posada

**Affiliations:** 1 Laboratorio de Biología Sintética, Estructural y Molecular, Universidad de Colima, Carretera Los Limones-Loma de Juárez, Colima 28627, Mexico; jcaraballo0@ucol.mx (J.A.-C.); bthvelazquez@hotmail.com (B.V.-C.); lauespinosaba@gmail.com (L.E.-B.); overgara0@ucol.mx (O.L.-V.); 2 Facultad de Ciencias, Universidad de Colima, Bernal Díaz del Castillo 340, Colima 28045, Mexico; rsaenz@ucol.mx; 3 Departamento de Ciencias Químico-Biológicas, Universidad de Sonora, Hermosillo 83000, Mexico; alexis.lopez@unison.mx; 4 Consejo Nacional de Humanidades, Ciencias y Tecnologías, Laboratorio de Biología Sintética, Estructural y Molecular, Universidad de Colima, Carretera Los Limones-Loma de Juárez, Colima 28627, Mexico; ycardenas11@ucol.mx; 5 Consejo Nacional de Humanidades, Ciencias y Tecnologías, Centro de Nanociencias y Nanotecnología, Universidad Nacional Autónoma de México, Km 107 CarreteraTijuana-Ensenada, Ensenada 22860, Mexico; azarate@ens.cnyn.unam.mx; 6 Facultad de Ciencias Biológicas y Agropecuarias, Universidad de Colima, Autopista Colima-Manzanillo, Tecomán 28100, Mexico; osuna_juan@hotmail.com; 7 Centro Universitario de Investigaciones Biomédicas, Universidad de Colima, Avenida 25 de julio 965, Colonia Villa de San Sebastián, Colima 28045, Mexico; ebonales0@ucol.mx

**Keywords:** carbohydrate metabolism, CM-CD, MdxE, SBP, starch-converting pathway

## Abstract

Extracellular synthesis of functional cyclodextrins (CDs) as intermediates of starch assimilation is a convenient microbial adaptation to sequester substrates, increase the half-life of the carbon source, carry bioactive compounds, and alleviate chemical toxicity through the formation of CD-guest complexes. Bacteria encoding the four steps of the carbohydrate metabolism pathway via cyclodextrins (CM-CD) actively internalize CDs across the microbial membrane via a putative type I ATP-dependent ABC sugar importer system, MdxEFG-(X/MsmX). While the first step of the CM-CD pathway encompasses extracellular starch-active cyclomaltodextrin glucanotransferases (CGTases) to synthesize linear dextrins and CDs, it is the ABC importer system in the second step that is the critical factor in determining which molecules from the CGTase activity will be internalized by the cell. Here, structure-function relationship studies of the cyclo⁄maltodextrin-binding protein MdxE of the MdxEFG-MsmX importer system from *Thermoanaerobacter mathranii* subsp. *mathranii* A3 are presented. Calorimetric and fluorescence studies of recombinant MdxE using linear dextrins and CDs showed that although MdxE binds linear dextrins and CDs with high affinity, the open-to-closed conformational change is solely observed after α- and β-CD binding, suggesting that the CM-CD pathway from Thermoanaerobacterales is exclusive for cellular internalization of these molecules. Structural analysis of MdxE coupled with docking simulations showed an overall architecture typically found in sugar-binding proteins (SBPs) that comprised two N- and C-domains linked by three small hinge regions, including the conserved aromatic triad Tyr193/Trp269/Trp378 in the C-domain and Phe87 in the N-domain involved in CD recognition and stabilization. Structural bioinformatic analysis of the entire MdxFG-MsmX importer system provided further insights into the binding, internalization, and delivery mechanisms of CDs. Hence, while the MdxE-CD complex couples to the permease subunits MdxFG to deliver the CD into the transmembrane channel, the dimerization of the cytoplasmatic promiscuous ATPase MsmX triggers active transport into the cytoplasm. This research provides the first results on a novel thermofunctional SBP and its role in the internalization of CDs in extremely thermophilic bacteria.

## 1. Introduction

Carbohydrate metabolism via cyclodextrins (CM-CD) is an unusual microbial pathway that implies carbon assimilation from starch through the synthesis of CDs as intermediates [1,2,3]. CDs are toroidal-shaped α-(1,4)-linked oligosaccharides commonly composed of six to eight glucopyranose units (named α-, β- and γ-CDs, respectively) [4]. Although the synthesis of CDs occurs by the cyclization of glucosyl-intermediates from the starch substrate by the catalysis of extracellular cyclomaltodextrin glucanotransferases (CGTases; EC 2.4.1.19) from subfamily 2 of the glycoside hydrolase family 13 (GH13_2) [5,6], CGTases can also yield fermentable sugars and linear dextrins through hydrolysis and disproportionation activities [7]. Since CDs are functional amphipathic molecules with higher heat-resistant values than linear dextrins, the ability of microorganisms to circularize starch to produce CDs not only allows substrate sequestration but also increases the half-life of the carbon source and bioavailability [8], making them capable of hosting, transporting, and solubilizing nonpolar guest molecules to alleviate the chemical toxicity of damaging compounds [9,10,11] or in contrast to carrying beneficial bioactive molecules [12]. Hence, the CM-CD pathway is a convenient microbial adaptation to compete for resources and to survive in adverse environments [3,11,13].

The CM-CD pathway was proposed early for the CD-producer mesophilic Gram-negative (G−) *Klebsiella oxytoca* [1,14], showing that it actively internalizes CDs across the microbial membrane through a sugar ABC importer system. Further descriptions for similar ABC importer systems from mesophilic Gram-positive (G+) Bacilli class bacteria [2] and hyperthermophilic Thermococci class archaea [15] are consistent with the formally established sugar type I ATP-dependent ABC importer systems [16] and functionally comparable with maltose (G2) ABC importer, MalEFGK_2_, from *Escherichia coli* [17]. Accordingly, CD internalization comprises a transmembrane complex formed by two permease subunits, MdxFG, named CymFG, CgtDE, and YvfL-YvfM in *K. oxytoca*, *Thermococcus* sp., and *Bacillus subtilis*, respectively [2,14,15]. The translocation of CDs into the cytoplasm via the MdxFG transmembrane complex is triggered by a dedicated MdxX ATPase in G− bacteria (CymD in *K. oxytoca*) [14] or by a promiscuous MsmX ATPase in G+ bacteria and archaea [2,15,18]. Although the mechanism by which CDs pass through the peptidoglycan (PG) layer from G+ is unknown, it has been shown that passive diffusion through the outer membrane occurs via a β-barrel CD porin channel (CP; CymA) in G− *K. oxytoca*, which selectively transports bulky CDs to the periplasm of G− [19].

Once CDs pass to the periplasm, the sugar-binding protein (SBP) MdxE detects, binds, and delivers the cyclo/maltodextrin molecules to the MdxFG-(X/MsmX) importer system [14,20,21]. While MdxE from G+ is anchored to the cytoplasmic membrane outer surface via an N-terminal lipid moiety covalently bound to a Cys residue [22], in G−, MdxE is an untethered component of the periplasm [14,23]. The crystallographic structure of the cyclo/maltodextrin-binding protein MdxE from G+ *Thermoactinomyces vulgaris* (*Tvu*CMBP, PDB ID: 2ZYK) showed the classical architecture of bacterial SBPs [21,24,25], consisting of two N- and C-domains joined by hinge regions with a sugar-binding site for CDs and linear dextrins. Nevertheless, despite the binding of linear dextrins and CDs, *Tvu*CMBP only forms productive complexes with CDs, switching from an open to a closed form to deliver ligands into the MdxFG permeases [21], simulating a Venus flytrap mechanism. While obtaining the closed conformation is a requirement for MdxFG components to recognize the MdxE-CD complex [21,26], a semiclosed/open form of MdxE prevents unspecific sugars from being internalized by the cell [27]. Nonetheless, the structural keys governing the recognition of the MdxE-CD complex by the MdxFG-(X/MsmX) importer system to translocate CDs into the cytoplasm remain obscure.

Recently, we reported the entire CM-CD pathway from extremely thermophilic bacteria (T_opt_ ≥ 70 °C) by exploring ~246 (meta)genomes from microbial communities living in a wide variety of hot environments on Earth [13]. Sequence analysis revealed that *Caldanaerobacter subterraneus* ssp., *Thermoanaerobacter* spp., and *Thermoanaerobacterium* spp. encoded an exceptional gene cluster of ~30 genes (named *cld*, *thm*, and *thb*, respectively) that encrypts the proteins related to the four steps of the CM-CD pathway from Thermoanaerobacterales, involving synthesis, transportation, degradation, and metabolic assimilation of CDs from starch. In the first step, extracellular thermophilic three-domain CGTases convert the surrounding starch substrate to α-, β-, and γ-CDs, as well as linear dextrins and fermentable sugars [13]. According to the sequence analysis of proteins encoded in the *cld*/*thm*/*thb* gene clusters [13], while degradation of CDs in the third step of the pathway occurs in the cytoplasm by the action of the functionally characterized cyclodextrinase (CDase), α-glucan phosphorylase (GP), and glucoamylase (GA) enzymes [28,29,30], the metabolic assimilation in the fourth step follows the typical Embden–Meyerhof–Parnas (EMP) glycolytic pathway, which includes a phosphoglucose isomerase (Pgi), 6-phosphofructokinase (PfkA) and the functionally characterized pyruvate kinase (PykF) [13,31]. Sequence analysis also showed that in the second step of the CM-CD pathway, a *mdx*EFG cassette that is adjacent to the CGTase-encoding gene, as well as a promiscuous ATPase, MsmX, distally located from the *cld*/*thm*/*thb* gene clusters, complete the entire putative cyclo/maltodextrin ABC importer system, MdxEFG-MsmX, from the Thermoanaerobacterales order [13]. Similar to the sulfur-reducing hyperthermophilic Themoccoci archaea [15,32,33], the synthesis of functional CDs as intermediates of the CM-CD pathway appears to be significant in Thermoanaerobacterales to thrive under extremophilic starch-poor environments [13].

Here, structure-function relationship studies of the novel cyclo/maltodextrin-binding protein MdxE from *Thermoanaerobacter mathranii* subsp. *mathranii* A3 (NCBI Taxonomy ID: 583358) are presented. Calorimetric and fluorescence studies of recombinant MdxE using linear dextrins and α-, β-, and γ-CDs showed that the open-to-closed conformational change is solely triggered after α- and β-CD binding. Experimental confirmation of MdxE-(α-/β-CD) complex formation, along with docking simulations, homology modeling, and structural bioinformatic analysis of the entire CD MdxFG-MsmX importer system from this extremely thermophilic bacterium, revealed the structural keys that appear to be involved in the binding, internalization, and delivery mechanisms of a novel and intriguing thermophilic ABC importer system for functional CDs.

## 2. Results and Discussion

### 2.1. MdxE Production and Purification 

**Figure 1:**
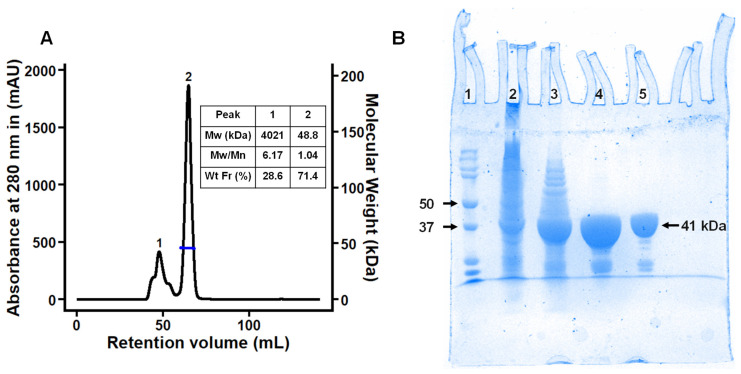
Purification of recombinant MdxE. (**A**) SEC-DLS coupled experiment of MdxE. Right inset: molecular weight (Mw), polydispersity index (Mw/Mn), and weight fraction (Wt Fr) of chromatographic peaks 1 and 2. Note that the MdxE aggregates (4021 kDa, peak 1) correspond to 28.6% of the total injected sample, showing the tendency of MdxE to form aggregates. (**B**) Coomassie Blue-stained SDS-PAGE gel (12%). Lane 1, molecular-weight markers (Bio-Rad, labeled in kDa). Lane 2, insoluble fraction of MdxE production. Lane 3, soluble fraction of MdxE production after a heat treatment procedure (60 °C, 20 min). Lane 4, purified MdxE after Ni^2+^-affinity chromatography. Lane 5, purified recombinant MdxE with optimal monodispersity (Mw/Mn = 1.04, peak 2) after SEC-DLS analysis.

To evaluate the binding affinities for linear dextrins and CDs, as well as the associated open-to-closed conformational changes of SBP MdxE, from *T. mathranii* subsp. *mathranii*, a recombinant form was successfully produced in *E. coli* SHuffle T7. The truncated form of MdxE consists of 380 residues with a calculated molecular mass of 41.5 kDa, including a C-terminal His6-tag sequence without the first 24 residues (Met1-Gly24) of the signal peptide (SP) and the N-terminal intrinsically disordered region (Cys25-Pro46) adjacent to the SP. Hence, to facilitate the recombinant production and functional characterization of MdxE, the conserved Cys25 residue crucial to anchoring the SBPs from G+ to the cytoplasmic membrane outer surface through a covalently bound lipid moiety was deleted in the truncated form [13,34]. Protein purification was performed in three sequential steps: heat treatment, nickel-affinity chromatography, and size-exclusion chromatography (SEC)-dynamic light scattering (DLS) coupled experiments (Figure 1A), yielding ~13 mg of purified MdxE per liter of culture according to the final Bradford quantification assay. The major peak collected after SEC-DLS analysis corresponding to 71.4% of the sample injected showed a calculated polydispersity index (M_w_/M_n_) of 1.04 and a molecular mass of 48.8 kDa (see the inset in Figure 1A), indicating that the biological assembly of MdxE is monomeric (peak 2). The electrophoretic profile in Figure 1B shows that recombinant MdxE was successfully purified and lies between 37–50 kDa bands, which was expected according to MdxE calculated molecular mass.

### 2.2. Homology Modeling of MdxE

**Figure 2:**
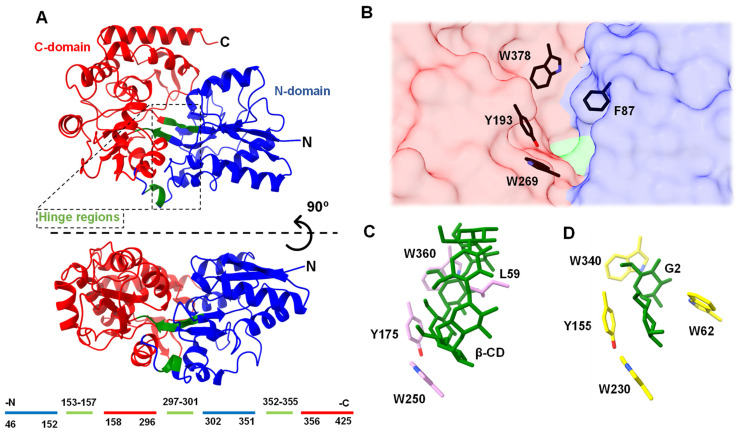
(**A**) Homology model of MdxE showing the N–domain (blue), C–domain (red), and hinge regions (green) in two views related by a horizontal rotation of 90 degrees. The residues comprising the N–domain, C–domain, and hinge regions are indicated below the MdxE model, following the same color code. The conserved aromatic triad involved in sugar recognition in the C–domain (red molecular surface) and the hydrophobic residue in the N–domain (blue molecular surface) involved in obtaining the closed form are shown in (**B**) MdxE (black cylinders), (**C**) *Tvu*CMBP (cyan cylinders) in complex with β–CD (PDB ID: 2ZYN, [36], and (**D**) *Eco*MBP (yellow cylinders) in complex with G2 (PDB ID: 1ANF) [37,38,39].

To obtain insights into the structural basis of MdxE, homology modeling was applied to build the three-dimensional (3D) structure (Figure 2). The overall architecture of MdxE comprises two globular N- and C-domains, as typically found in SBPs [21,24,35]; both domains consist of a β-sheet core surrounded by α-helices linked by hinge regions that allow switching from an open (sugar-free or nonfunctional sugar-bound) to a closed form (functional sugar-bound) for cellular internalization [21,26]. Structural comparisons of MdxE with the maltodextrin-binding protein from *Alicyclobacillus acidocaldarius* (*Acy*MBP, PDB ID: 1URD) [35], which superpose 357 C^α^ with MdxE with an r.m.s.d. of 0.22 Å, allowed the identification of residues involved in the different interactions of both domains. Thus, while the N-domain consists of 46–152 and 302–351 residues, the C-domain consists of 158–296 and 306–425 residues. Hinge regions located at the bottom of the sugar-binding site of MdxE and comprising three small segments, 153–157, 297–301, and 352–355 residues, were identified by structural comparisons with *Tvu*CMBP (PDB ID: 2ZYN) [36], which superpose 320 C^α^ with MdxE with an r.m.s.d. of 2.2 Å.

As seen in other SBPs from bacteria, the MdxE homology model showed the conserved aromatic triad Tyr193, Trp269, and Trp378 in the C-domain (Figure 2B) that recognizes different sugars through CH-π interactions [36,38]. Accordingly, structural comparisons revealed that the aromatic triad in MdxE is identical to the aromatic triad Tyr175/Trp250/Trp360 in *Tvu*CMBP (Figure 2C) and Tyr155/Trp230/Trp340 in the well-studied maltodextrin-binding protein from *E. coli* (*Eco*MBP, PDB ID: 1ANF) [39], Figure 2D), strongly suggesting that it has an essential role in sugar recognition. Conversely, the N-domain face at the sugar-binding cleft of MdxE exhibits the aromatic Phe87 residue (Figure 2B) at the same position as Leu59 and Trp62 in *Tvu*CMBP and *Eco*MBP, respectively (Figure 2C,D) [36,39], which have been proposed to play an important role in adopting the closed form in (*Tvu*CMBP/*Eco*MBP)-ligand complex formation. Hence, while Leu59 in *Tvu*CMBP orients toward the central cavity of CDs for its stabilization [21,36], Trp62 is essential for G2 accommodation in the sugar-binding site of *Eco*MBP (PDB ID: 1ANF) [37,39]. A similar mechanism has been reported for CGTases, in which a central aromatic residue is crucial for CD formation (cyclization activity) [40,41], as well as cyclodextrinases (CDases), in which an aromatic residue is critical in stabilizing CDs at the active site for hydrolytic activity [21,28]. This information suggests that MdxE has a typical sugar-binding site contour specifically adapted to bind and deliver cyclo/maltodextrins to MdxFG permeases in the second step of the CM-CD pathway from *T. mathranii* subsp. *mathranii*.

### 2.3. Determination of MdxE Binding Affinities 

**Figure 3:**
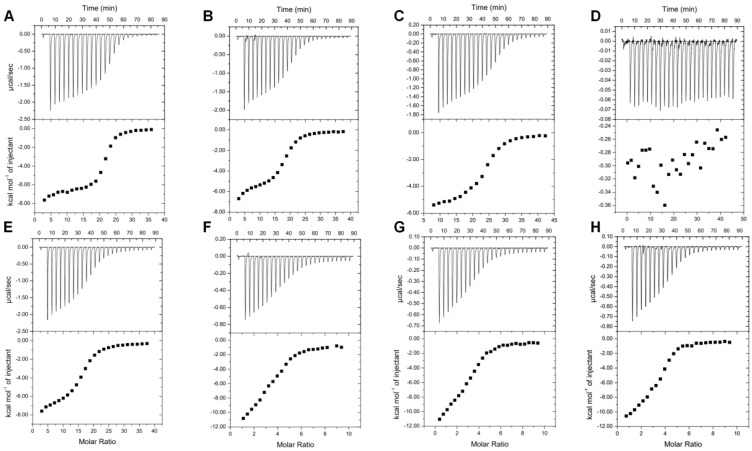
ITC studies of MdxE titrated with (**A**) α−CD, (**B**) β−CD, (**C**) γ−CD, (**D**) maltose (G2), (**E**) maltotriose (G3), (**F**) maltopentaose (G5), (**G**) maltohexaose (G6), and (**H**) maltoheptaose (G7).

To assess the binding affinities of MdxE toward different linear dextrins and CDs for the CM-CD pathway, functional experiments of MdxE-ligand complex formation were performed using isothermal titration calorimetry (ITC) to determine the dissociation constant (*K_d_*) and enthalpy (ΔH) for ligand binding, as well as the Gibbs free energy (ΔG) contribution to the binding (Figure 3). 

As summarized in Table 1, MdxE showed the highest affinity toward α-CD, with a *K_d_* value of 0.61 μM. Nevertheless, although the *K_d_* values for β-CD and γ-CD were 2.04 and 1.51 μM, respectively, the affinity of MdxE for linear dextrins was directly proportional to the polymerization degree (DP). Hence, while the *K_d_* value for G7 was 0.91 μM, the affinity of MdxE for G3 was much lower (*K_d_*, 3.02 μM), and no binding interaction was observed for G2 (Table 1). In fact, despite titration with G2 resulting in short heat pulses, no sigmoidal model could be fitted (Figure 3), suggesting very low affinity or no binding interaction between MdxE and G2. This behavior contrasts with the results observed for *Tvu*CMBP, *Acy*MBP, and *Eco*MBP, which showed a high affinity toward G2 [21,22,39]. However, a similar finding has been reported for CD-binding protein from *K. oxytoca* (CymE) since the quenching effect of G2 during fluorescence assays to determine *K_d_* was solely marginal compared to CDs and linear dextrins with high DP [14]. Accordingly, since extracellular three-domain ABC CGTases involved in the first step of the CM-CD pathway from Thermoanaerobacterales recycle G2 for disproportionation activity to produce linear dextrins with a DP > 3 [7,13], the latter observations suggest that the inability of MdxE to bind G2 might be related to the activation of a different internalization pathway than CM-CD for residual G2. Indeed, G2, G3, and α-trehalose are internalized by the same catabolite-repressible ABC importer system in *Thermoanaerobacter ethanolicus* [42]. Similarly, *K. oxytoca* and *B. subtilis* express an independent maltose/maltodextrin (*mal*) system for G2 internalization, as the CM-CD represents a secondary pathway for these microorganisms [14,33,43,44]. In agreement, the genome of *T. mathranii* subsp. *mathranii* encodes three putative ABC sugar importer systems (Figure S1), one of which includes a putative SBP (NCBI ID: WP_013149854.1) that shares ~50% sequence identity with the characterized G2-binding protein from *Thermotoga maritima* (tmMBP, PDB ID: 6DTQ) [45].

Compared to MdxE, similar *K_d_* values for linear dextrins and CDs have also been determined for various SBPs (Table 1). Nevertheless, except for G2, the ITC analysis cannot determine whether MdxE adopts the open form with a nonfunctional sugar-bound (unproductive complex) or the closed form with the sugars listed in Table 1. An initial approach would be inferring that the magnitude of the binding affinities observed for MdxE in Table 1 is directly proportional to the probability of switching from an open to a closed form to deliver ligands into the MdxFG permeases from the ABC importer system. Nevertheless, the crystallographic structures of *Tvu*CMBP and *Eco*MBP reveal the opposite [36,39,46]. For instance, although the *K_d_* values of *Eco*MBP are identical for G2 and β-CD (Table 1), *Eco*MBP complexed with G2 adopts the closed form (PDB ID: 1ANF, Figure 2D) [39], while the open form is observed when complexed with β-CD (unproductive complex, PDB ID: 1DMB, [46]. Likewise, although the *K_d_* values of *Tvu*CMBP are similar for linear dextrins and CDs (Table 1), *Tvu*CMBP complexed with α-, β-, and γ-CDs adopts the closed form (PDB IDs: 2ZYM, 2ZYN, 2ZYK, Figure 2C) [21,36], whereas the open form is observed when complexed with linear dextrins (unproductive complex, PDB ID: 2ZYO) [36], despite β-CD even showing lower affinity (*K_d_* = 1.2 μM) than linear dextrins. Hence, although ITC experiments confirm when an SBP has no inherent ligand-binding capacity, such as MdxE with G2 (Figure 3), additional experiments are required to determine the functional dynamics associated with binding specificity.

### 2.4. Determination of the Open-to-Closed Conformational Change by MdxE

**Figure 4:**
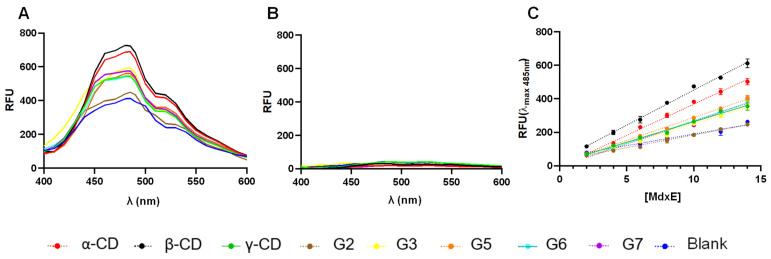
Fluorescence determination of the open-to-closed conformational change by MdxE complexed with CDs and linear dextrins. Note that the scans were taken in the 400–600 nm range. (**A**) MdxE-ligand complexes (16 μM) bound to ANS. (**B**) Ligands with ANS (blank). (**C**) The increasing intensity was monitored by relative fluorescence unit (RFU) measurements at λ_max_ = 485 nm as a function of MdxE-ligand concentration (2–14 μM). Note that each H_o_ value is obtained as the slope of each of the linear functions [47].

To evaluate the switching from an open to a closed form of productive MdxE-ligand complexes obtained with the sugars listed in Table 1, changes in the protein surface hydrophobicity (H_o_) of MdxE-ligand complexes using the fluorescent probe 8-anilinonaphthalene-1-sulfonate (ANS) were determined (Figure 4). Since H_o_ is governed by the slope generated as the fluorescence response changes upon binding interaction [47,48], increasing MdxE concentrations (2–14 µM) inside a linear range (Figure S2) were tested using an excess of ligand (350 µM) at a fixed concentration of ANS (20 µM) to assure that MdxE was in its ligand-saturated state based on ITC studies (Figure 3). First, a notable change in fluorescent response at λ_max_ of 485 nm was observed with increasing MdxE-ligand concentrations at a constant ANS concentration (10 μM) (Figure 4A). Since no effect on fluorescent response was observed between ANS and ligands (Figure 4B), this change could be attributed to the productive sugar-binding interactions that allowed obtaining a MdxE closed form. In fact, docking simulations of MdxE models in open and closed conformations with ANS (Figure S3) resulted in a striking difference in the total number of ANS molecules binding to MdxE in the open form compared to the closed form. Accordingly, while 18 ANS molecules were observed binding different hydrophobic sites on the MdxE surface in the closed form, only 11 probes were found in the open form. This observation indicates that only upon binding CDs and linear dextrins that the cell will functionally internalize does MdxE undergo a conformational change toward the closed form (functional sugar-bound), exposing more hydrophobic sites for ANS molecules and thus increasing H_o_. Notably, the slopes obtained from the linear fit model at λ_max_ = 485 nm in Figure 4C shows significant differences (*p* < 0.05) between the blank, MdxE-ANS (open form), and the CDs and linear dextrins tested, whose H_o_ value associated with the open-to-closed conformational change remains statistically invariant compared to the blank. As expected from the ITC analysis (Figure 3), no MdxE-G2 complex formation was observed by fluorescence measurements (Figure 4). The remaining CDs and linear dextrins tested were grouped according to H_o_ values for further statistical analysis (Table 2). 

According to Tukey’s multiple comparisons test (Table S1), there were no significant differences (*p* < 0.05) between the ligands of the first group, G3–G7/γ-CD, suggesting a similar conformational change as a function of H_o_ values that might be associated with an inactive semiclosed/open form of MdxE-(G3–G7/γ-CD) complexes (Table 2). Nevertheless, the ligands of the second group, α- and β-CDs, placed in an upper region with significantly different H_o_ values (*p* < 0.05) compared to the first group, showed that they have functional effects by triggering the closed form of MdxE for cellular internalization. As expected, the H_o_ value for MdxE-G2 complex formation was close to that of the blank (Table 2), confirming that MdxE has no affinity for G2. Although the thermodynamic parameters determined by ITC analysis revealed that the formation of the MdxE-ligand complexes is an exothermic and spontaneous process, the fluorescence analysis showed that the open-to-closed conformational change is solely triggered by α- and β-CDs (Table 2). Since α- and β-CDs are the main cyclization products from the starch substrate of three-domain ABC CGTases from Thermoanaerobacterales [13], these results strongly suggest that the CM-CD pathway is exclusive for transportation, degradation, and metabolic assimilation of α- and β-CDs through the type I ABC MdxEFG-MsmX importer system.

### 2.5. Structural Basis for the Open-to-Closed Conformational Change of MdxE

**Figure 5:**
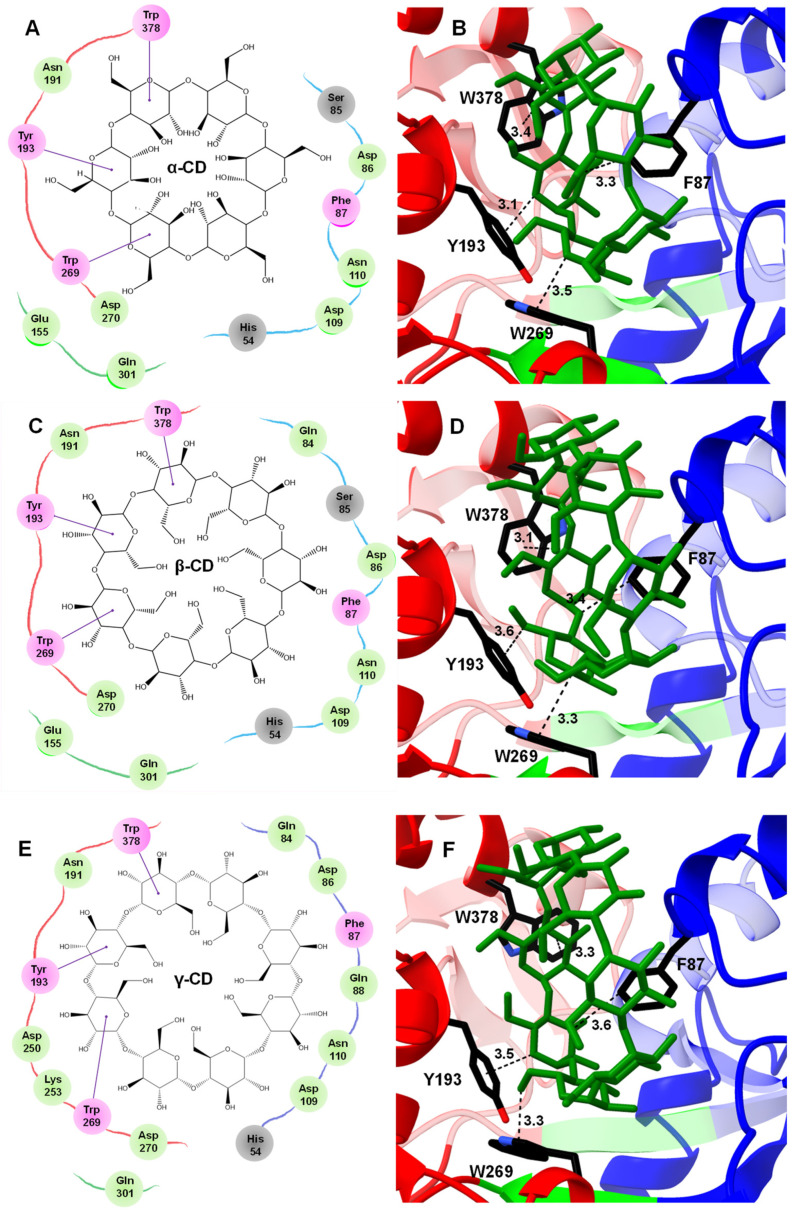
Docked structures of α-, β-, and γ-CDs in the sugar-binding site of MdxE. (**A**,**B**) MdxE/α-CD. (**C**,**D**) MdxE/β-CD. (**E**,**F**) MdxE/γ-CD. The two-dimensional (2D) interaction plots show the hydrogen bonds between glucose (G1) and the side chain of a residue in green, hydrophobic interactions in violet, and hydrogen bonds with the main chain atoms in gray. The 3D docked structures exhibit the key residues (black cylinders) in the N-domain (blue) and C-domain (red) involved in CD recognition. The residues in the 2D interaction plots are linked with red, blue, and green lines representing the C-domain, N-domain, and hinge regions, respectively. Note that the absence of Glu155 from hinge region I in the MdxE/γ-CD complex might affect obtaining the closed form. Distances are in Å.

To identify the critical residues in MdxE that are involved in CD recognition for the CM-CD pathway, docking simulations of MdxE in the open and closed forms with α- and β-CDs (Figure 5, Table S2) were carried out using the coordinates of the MdxE model as a template for calculations (Table S3). Both docked structures MdxE/α-CD (Figure 5A) and MdxE/β-CD (Figure 5B) in the closed form showed the CDs stabilized by the conserved aromatic triad Tyr193, Trp269, and Trp378 in the C-domain and resting on top of the aromatic Phe87 residue in the N-domain with binding ΔG values of −10.8 and −9.7 kcal mol^−1^, respectively (Table S2). This latter observation correlates with the functional studies for α- and β-CDs (Figure 3 and Figure 4), suggesting an energetically favorable CD binding by MdxE in the closed form to deliver them into the MdxFG permeases from the ABC importer system.

Regarding the residues from the N- and C-domains involved in the CD-binding site of MdxE, as well as hinge regions at the bottom of the cleft (Figure 5), the docking simulations showed the pair Asn191/Asp270 from the C-domain forming hydrogen bonds with α-, β-, and γ-CDs and the aromatic triad Tyr193/Trp269/Trp378 forming hydrophobic interactions with three contiguous G1 from the three CDs. Owing to the differences in the CD size, while the hydrogen bonds between α-, β-, and γ-CDs and Asp86/Asp109/Asn110 from the N-domain are conserved, the hydrogen bond between Gln84 and α-CD is absent, and a hydrogen bond between Gln88 and γ-CD is gained. Likewise, although the hydrogen bond between α-, β-, and γ-CDs and the main chain of His54 is conserved, the hydrogen bond formed by Ser85 was solely observed for α- and β-CDs, but two hydrogen bonds between γ-CD and Asp250/Lys253 were gained. Furthermore, docking simulations of MdxE in the open and closed forms with linear dextrins were also performed (Figure S4). Thus, while acceptable binding ΔG values for the closed form of MdxE/(G3–G7) complexes were found (Table S2), fewer interactions than those observed for CDs (Figure 5) between the linear dextrins and the residues of the sugar-binding site were found, suggesting the formation of unproductive complexes between MdxE and linear dextrins that display inactive semiclosed/open forms (Figure S4, Table S2).

**Figure 6:**
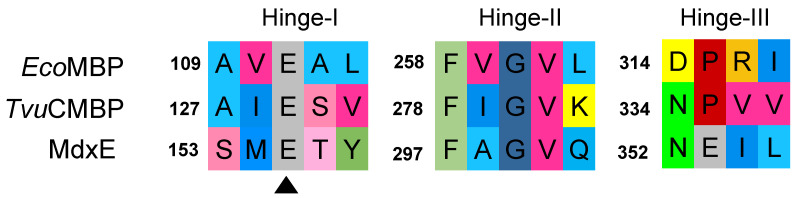
Amino acid sequence alignment of *Eco*MBP, *Tvu*CMBP, and MdxE. Note the conserved Glu residue (black triangle) in hinge region I of SBPs. Sequence alignment was performed using ClustalW [49].

Nevertheless, since subtle structural changes in the hinge regions of MdxE-ligand complexes were observed, special attention was focused on the Venus flytrap hinge-bending motion of MdxE by comparing the open and closed forms of all docked structures (Table S2). Structural analyses revealed that Glu155 and Gln301 from hinge regions I and II, respectively (Figure 6), form concerted hydrogen bonds with solely α- and β-CDs (Figure 5A,C), suggesting that they act together to provide part of the driving force to ensure the bending of MdxE and the formation of the closed form. Accordingly, the dynamics of the open-to-closed conformational changes of *Tvu*CMBP crystal structures revealed that the critical factor of this reorganization is centered on the motion of hinge regions I and II at the bottom of the sugar-binding site [36]. Strikingly, amino acid sequence alignment between the hinge regions of *Tvu*CMBP, *Eco*MBP, and MdxE showed that Glu155 of MdxE (Figure 5) is found at the exact position as the conserved Glu111 and Glu129 from hinge region I of *Eco*MBP and *Tvu*CMBP, respectively, which is involved in adopting the closed form of (*Tvu*CMBP/*Eco*MBP)-ligand complexes (Figure 6) [36]. Although Gln301 was also observed to form hydrogen bonds with γ-CD (Figure 5E) and G3/G5/G7 (Figure S4) in the docking simulations of the closed forms of MdxE, the conserved Glu155 is exclusive for MdxE/(α-/β-CD) complexes, strongly suggesting its structural role in the open-to-closed conformational changes of MdxE. Nevertheless, future X-ray crystal structure determination, site-specific mutants, and functional studies are needed to determine the function of Glu155 in MdxE.

### 2.6. Structural Insights into the Internalization Mechanism of CDs from Thermoanaerobacterales

**Figure 7:**
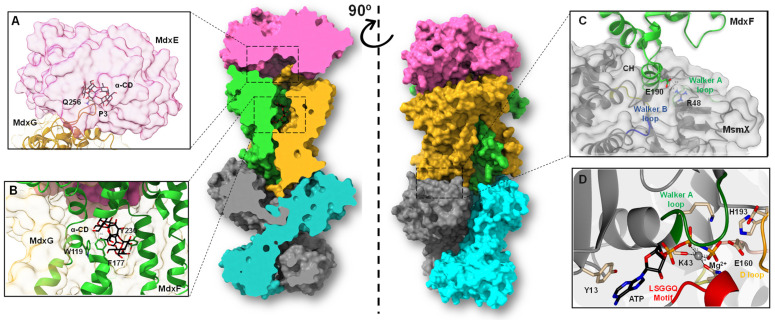
Homology model of the entire type I ABC importer system, MdxEFG-MsmX, from *T. mathranii* subsp. *mathranii*. (**A**) Close-up of the binding site showing MdxE on the magenta molecular surface, the spoon loop (P3) of MdxG in goldenrod ribbon representation, and α-CD in black and red cylinders. Note that Gln256 from MdxG seems to be involved in removing the CDs from the MdxE-binding site. (**B**) Close-up of the binding pocket showing MdxF and MdxG in green and goldenrod ribbon representations, respectively, the aromatic triad Trp119/Phe177/Tyr230 in green cylinders, and α-CD in black and red cylinders. (**C**) Close-up of CH from MdxF (green) showing the conserved Glu190 in green and red cylinders, directly interacting with Arg48 in gray and blue cylinders from the Walker A (green) motif in MsmX. The Walker B motif is shown in deep blue. The NBD subunit is shown on a gray molecular surface. (**D**) Close-up of the detailed interactions at the active side of the NBD with a docked ATP molecule. The MsmX subunit is shown in gray ribbon representation, as well as signature motifs LSGGQ (red), Walker A (dark green), and D-loop (orange), which along with the Walker B motif, encompass most of the residues (Tyr13, Lys43, Glu160, and His193) needed for ATPase activity and signal transmission between the TMD-NBD. Note that the interaction between Lys43 and the coordinated Mg^2+^ atom (gray sphere) is crucial for the cleavage of γ-phosphate from the ATP molecule.

To propose an internalization mechanism for α- and β-CDs by the CM-CD pathway from Thermoanaerobacterales, homology modeling was applied to build the 3D structures of the extracellular CGTase and the entire MdxEFG-MsmX importer system (Figure 7). Structural comparisons of the putative extracellular three-domain CGTase from *T. mathranii* subsp. *mathranii* (ThmA) with the crystallographic structure of the three-domain CGTase from *C. subterraneus* (CldA, PBD ID: 6WNI, sequence identity: 83%), which superposes 437 C^α^ with ThmA with an r.m.s.d. of 0.40 Å, allowed the identification of the central aromatic residue Phe218, the catalytic triad Asp252/Glu281/Asp353, and the hydrophobic clamp Met283/Trp206 in ThmA, which are essential for producing CDs and linear dextrins from the starch substrate [7,13]. The entire model of the ABC MdxEFG-MsmX importer system from Thermoanaerobacterales (Table S3, Figure 7) was built upon the crystallographic structure of MalEFG-MalK from *E. coli* (OF, PDB ID: 3PUV) [50], which superposes 938 C^α^ with MdxEFG-MsmX with an r.m.s.d. of 2.9 Å, as it is the only sugar type I ABC importer system well studied thus far. Several residues of MdxEFG-MsmX involved in ligand translocation across the microbial membrane were identified. Accordingly, the spoon loop (P3) in the permease MdxG containing the conserved Gln256 that seems to be involved in removing α- and β-CDs from the MdxE-binding site was found at the exact position as the conserved Gln256 from MalG (PDB ID: 3PUV, Figure 7A) [50,51]. Likewise, the aromatic triad Trp119/Phe177/Tyr230 in the permease MdxF, which stabilizes the α- and β-CDs through CH-π interactions (Figure 7B), was identified by structural comparisons with the homologous subunit MalF (PDB ID: 3PUV) [51]. Moreover, the coupling helixes (CH) in MalFG, responsible for the transmembrane domain–nucleotide-binding domain (TMD-NBD) coupling that regulates the conformational changes triggered by ATP hydrolysis [52], were identified in MdxFG (Figure 7C). Additionally, Glu190 in both subunits of MdxFG was found to make an electrostatic interaction with Arg48 in the active site of NBDs (Figure 7C), suggesting that the interaction could be correlated to the dimerization of the cytoplasmatic promiscuous ATPase, MsmX, as previously found in MalEFG-MalK [53]. Finally, the conserved LSGGQ motif, Walker A and B, as well as several motifs involved in ATP hydrolysis by MsmX (Figure 7D) [16,18], were identified by structural comparisons with MalK (PDB ID: 1Q12) [54], which superposes 712 C^α^ with an r.m.s.d. of 0.51 Å.

**Figure 8:**
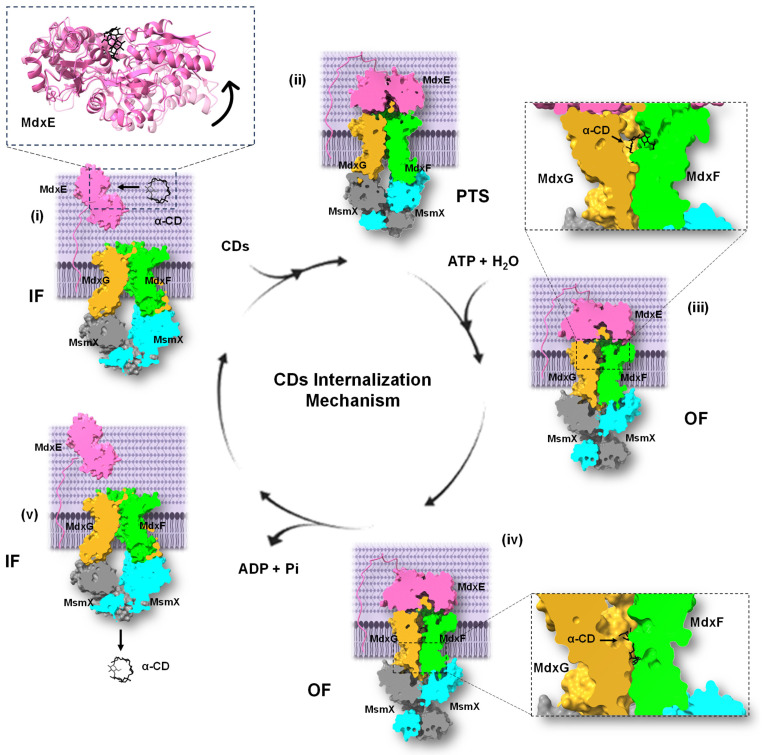
Proposed internalization mechanism of the type I ABC importer system, MdxEFG-MsmX, from Thermoanaerobacterales. The mechanism encompasses five main steps: (**i**) Ligand recognition by MdxE. (**ii**) Coupling of MdxE to the MdxFG-MsmX core unit and transition into a PTS. (**iii**) Ligand transfer into the MdxFG transmembrane channel via an OF conformation obtained by MsmX dimerization. (**iv**) The dimerization process is completed, and ATP hydrolysis occurs, releasing MdxE from the core unit, ADP and Pi. (**v**) The ligand is finally translocated into the cytoplasm, allowing transition into an IF conformation for the next MdxE cycle. The three states of MdxEFG-MsmX from Thermoanaerobacterales were built upon MalEFG-MalK structure from *E. coli* (PDB IDs: 3FH6, 3PUZ, and 3PUV) [50,51,55].

Hence, once α- and β-CDs have entered the PG layer from G+ bacteria, the SBP MdxE, which is anchored to the cytoplasmic membrane outer surface by a lipidic moiety, binds the CD molecule through an open-to-closed conformational change that releases it into the transmembrane channel formed by MdxFG. However, releasing molecules into the cytoplasm requires ATP-mediated conformational changes of the MdxFG-MsmX complex. Therefore, MdxE couples onto the permease subunit MdxFG, triggering a pretranslocation state (PTS) to induce the dimerization of the cytoplasmatic ATPase MsmX, obtaining the active form that hydrolyzes an ATP molecule to initiate internalization [52]. This catalytic process triggers a mechanism in which MdxFG-MsmX adopts an outward-facing (OF) conformation where a periplasmic section of this unit will be open, allowing entry of the ligand into the channel through the conserved Gln256 of MdxG, which acts as a spoon to remove it from the sugar-binding site of MdxE (Figure 7A). The following interaction of CD with MdxFG involves the aromatic triad of the sugar-binding site of MdxF (Figure 7B), similar to the aromatic triad of MdxE (Figure 2B). Hence, the CD is trapped in the transmembrane channel before being released into the cytoplasm. Finally, MdxFG-MsmX returns to the initial inward-facing (IF) conformation, releasing the CD molecule into the cell for the next MdxE cycle (Figure 8).

## 3. Materials and Methods

### 3.1. Gene Cloning and Production of Recombinant MdxE

The *E. coli* codon-optimized N-terminally truncated form of MdxE (NCBI ID: WP_013150585.1) [13] was synthesized by Integrated DNA Technologies (Coralville, IA, USA) and subcloned into a pET-22b(+) expression vector (Novagen, Madison, WI, USA) between the *Nde*I and *Not*I restriction sites by Catálisis Biotechnology Company (Morelos, Mexico), resulting in plasmid pMdxE. Restriction analysis and DNA sequencing confirmed the synthesized insert with a sequence coding for six histidines at the C-terminus. The plasmid pMdxE was transformed into *E. coli* SHuffle T7 competent cells (New England BioLabs) by heat shock. Transformed cells were plated onto Luria–Bertani (LB) agar plates supplemented with 200 µg mL^−1^ ampicillin at 37 °C. Individual clones of SHuffle T7 harboring pMdxE were cultured in 5 mL LB medium overnight supplemented with 200 µg mL^−1^ ampicillin at 30 °C and then aliquoted into a sterile solution of 40% (*v*/*v*) glycerol and stored at −80 °C. For recombinant MdxE production, an overnight preculture (37 °C, 170 rpm) grown in 250 mL LB medium (200 µg mL^−1^ ampicillin) was used to inoculate 1 L 2xYT medium (200 µg mL^−1^ ampicillin) to an initial optical density at 600 nm (OD_600_) of ~0.1. The culture was incubated at 37 °C and 200 rpm until it reached an OD_600_ of ~0.6 and cooled to 22 °C, and expression was induced by adding a final concentration of 0.1 mM isopropyl β-D-1-thiogalactopyranoside (IPTG) to the medium. The induced culture was incubated for 12 h at 22 °C and 170 rpm before harvesting the cells by centrifugation (7500× *g*, 10 min, 4 °C). The pellet was resuspended in 10 mL of cold lysis buffer A (50 mM sodium phosphate pH 8.0, 500 mM NaCl, 2% (*v*/*v*) glycerol, 20 mM imidazole) supplemented with a half mini tablet of EDTA-free complete protease inhibitor cocktail (Roche Molecular Biochemicals) and 1 μg mL^−1^ DNAse to be disrupted by sonication on ice for 30 min with an amplitude of ~29%. The resulting solution was subjected to a heating step for 20 min at 60 °C to precipitate undesirable thermolabile *E. coli* proteins. Soluble proteins were separated from the insoluble fraction by centrifugation at 19,000× *g* for 1 h at 4 °C. The supernatant containing thermophilic His6-tagged MdxE was recovered and filtered using a 0.22 µm microfilter (Merck Millipore, Burlington, MA, USA).

### 3.2. Purification of MdxE and SEC-DLS Analysis

Recombinant MdxE was purified by two additional steps, including Ni^2+^-affinity chromatography and SEC-DLS coupled experiments using an ÄKTA Pure 25 M1 FPLC system with UNICORN v1.7.0.1 software (Cytiva, Marlborough, MA, USA) and a Zetasizer μV DLS instrument with OmniSEC v5.12 software (Malvern, UK), respectively. The filtered supernatant was loaded onto a 5 mL Ni^2+^-chelating HisTrap HP column (Cytiva) equilibrated with ten-bed volumes of buffer A. After washing with eight-bed volumes of buffer A to eliminate contaminants, bound MdxE was eluted with a linear gradient of the same buffer A but containing 500 mM imidazole. Elution fractions containing purified MdxE were collected and dialyzed against buffer B (50 mM Tris-HCl pH 7.5, 100 mM NaCl) using an ultrafiltration cell (Amicon Ultracel filter, 30 kDa molecular-weight cutoff, Merck Millipore). SEC-DLS coupled experiments were performed based on the method described previously [13]. A concentrated sample of MdxE at 10 mg mL^−1^ was filtrated using a 0.22 µm microfilter and resolved on a 120 mL HiLoad 16/600 Superdex 75 pg column (Cytiva) equilibrated with buffer B using a quartz flow cell of 8 μL (Malvern) and bovine serum albumin (BSA, Sigma-Aldrich, St. Louis, MO, USA) as a standard. A major peak corresponding to the MdxE monomer was collected, concentrated, and dialyzed against several volumes of buffer C (10 mM sodium phosphate pH 7.5) using a 30 kDa cutoff ultrafiltration cell for ITC and fluorescence experiments. While SDS-PAGE with Coomassie staining was also used to analyze the purity of MdxE, the protein concentration was determined by the Bradford assay using BSA as a standard [56].

### 3.3. ITC Measurements

Purified MdxE at 4 μM was titrated with 0.8 mM CDs and linear dextrins using a VP-ITC calorimeter (Cytiva) at 55 °C. In all measurements, the sample cell and syringe were filled with protein and ligand solution, respectively, and the reference cell was filled with ultrapure water. The stirring speed of the sample syringe during measurements was 250 rpm. After baseline stabilization and an initial injection of 2 µL, 25 successive injections of 11 µL were carried out with a 5 min equilibration period between injections. Since both protein and ligands were in buffer C, the heat of dilution was neglected in the experiments. The resulting data were analyzed in triplicate (*n* = 3) using the Origin 7 ITC analysis v7.0 software from Microcal by fitting the titration curves to a single binding site model.

### 3.4. Fluorescence Measurements

The changes in H_0_ associated with the open-to-closed conformational changes of productive MdxE-ligand complexes were determined based on the method described previously [47,57], using a Nanodrop ND-3300 fluorospectrometer (Thermo Fisher, Waltham, MA, USA) controlled by ND-3300 v2.8.0 software with ANS as a fluorescence probe at an excitation wavelength of 365 nm and 400–600 nm as the range for emission scan. The fluorescent response was measured in RFU using buffer C for all solutions. Different MdxE concentrations ranging from 2 to 14 µM were tested at fixed ANS concentrations of 20 µM and 350 µM ligand to guarantee saturation of MdxE based on ITC curves. For H_0_ measurements, MdxE-ligand complexes were heated at 55 °C for 10 min. Subsequently, ANS was added to form the (MdxE-ligand)-ANS complexes, and the solutions were heated again at 55 °C for 3 min; then, the solutions were cooled to room temperature. Data were analyzed in triplicate (*n* = 3), and H_0_ was estimated as the slope of the linear curve fitted for RFU versus MdxE concentrations plotted at the maximum emission wavelength (λ = 485 nm) using the emtrends() function from emmeans package in R [58,59]. A Tukey’s test (α = 0.05) for multiple comparisons was performed to determine significant differences in H_0_ between CDs and linear dextrins tested. All fluorescence measurements were made at 22 °C.

### 3.5. Homology Modeling and Docking Simulations 

Homology models of MdxE (NCBI ID: WP_013150585.1), MdxF (NCBI ID: WP_012995620.1), MdxG (NCBI ID: WP_012995619.1), and MsmX (NCBI ID: WP_012995743.1) from the sugar ABC importer system, as well as the three-domain ABC CGTase ThmA (NCBI ID: WP_013150586.1) [13], were built on the SWISS-MODEL server [60]. Accordingly, while MdxE models were built using the *Acy*MBP structure in the closed form (PDB ID: 1URD) [35] and the *Tvu*CMBP structure in the open form (PDB ID: 2ZYO) [36] as templates for calculations, ThmA, MdxF, MdxG, and MsmX models were built using the templates shown in Table S3. The entire model of the MdxEFG-MsmX importer system was assembled manually using Coot [61] upon the entire MalEFG-MalK structure from *E. coli* (OF, PDB ID: 3PUV) [50]. All the models were subjected to energy minimization using YASARA software v22.9.24 [62] and then validated using MolProbity [63]. Local docking simulations at the sugar-binding site of MdxE models with CDs and linear dextrins were performed using AutoDock Tools 1.5.4 and AutoDock Vina 1.1.0 [64]. Global docking simulations were also performed to visualize the differential binding of the ANS fluorescence probe to the MdxE models in open and closed conformations. All ligand structures were obtained from the PubChem database [65], transformed into 3D structures and minimized using Avogadro v1.95 [66]. The results of MdxE-ligand interactions were selected according to the best ΔG and *K*_d_ values for subsequent analysis. The molecular docking results were visualized using a BIOVIA discovery studio visualizer [67]. Structure analysis was carried out by manual inspection using UCSF Chimera [68] and Coot [61]. Illustrations were prepared using BIOVIA [67] and UCSF ChimeraX [68].

## 4. Conclusions

This work presents structure-function relationship studies of SBP MdxE from the sugar type I ABC importer system MdxEFG-MsmX of the CM-CD pathway from *T. mathranii* subsp. *mathranii*. Homology modeling and docking simulations revealed that MdxE possesses the conserved features commonly found in SBPs, such as two structural N- and C-domains linked by three small hinge regions located at the bottom of the sugar-binding site that allow the transition from an open to a closed conformation upon CD binding. While an aromatic triad Tyr193/Trp269/Trp378 in the C-domain recognizes and stabilizes the ligand through hydrophobic interactions, Phe87 protrudes from the N-domain and assists in stabilizing the CDs by inserting its side chain into the central cavity. ITC measurements showed that, except for G2, MdxE is capable of binding CDs and linear dextrins with high affinity and that the formation of MdxE-ligand complexes takes place in a spontaneous and exothermic interaction for every ligand tested. In contrast, fluorescence measurements and docking simulations showed that despite the binding of CDs and linear dextrins, the open-to-closed conformational change is solely triggered after α- and β-CD binding by the concerted action of hinge regions I and II. Structural bioinformatic analysis of the entire CD ABC importer system from *T. mathranii* subsp. *mathranii* also revealed the structural keys that appear to be involved in the binding, internalization, and delivery mechanisms of CDs by this nonclassical pathway from Thermoanaerobacterales, which is valuable in the competition for starch availability in extremophilic environments.

## Figures and Tables

**Table 1:** Comparison between *K_d_* values for MdxE and various SBPs determined by ITC measurements.

Ligand	*K_d_* (μM)				
	MdxE	*Tvu*CMBP *	*Eco*MBP *	*Acy*MBP *	CymE *
G2	ND	0.41	1.0	1.5	ND
G3	3.02	0.97	0.2	-	-
G5	2.44	0.2	-	-	-
G6	1.85	-	-	-	-
G7	0.91	-	-	-	70
α-CD	0.61	0.73	-	-	0.02
β-CD	2.04	1.2	1.0	-	0.14
γ-CD	1.51	0.23	-	-	0.3

* Data used here were obtained from the original publications [14,21,22,39]. ND = not detected.

**Table 2:** Summary of functional experiments of MdxE with different CDs and linear dextrins.

Ligand	Fluorescence Analysis		ITC Analysis		
	H_o_	R^2^	ΔH (kcal mol^−1^)	ΔG (kcal mol^−1^)	*K_d_* (μM)
α-CD	37.1 ^a^	0.989	−6.83	−7.21	0.61
β-CD	41.8 ^b^	0.992	−5.28	−5.82	2.04
γ-CD	24.5 ^c^	0.965	−6.50	−6.90	1.51
G3	24.3 ^c^	0.981	−8.64	−8.50	3.02
G5	27.3 ^c^	0.994	−13.94	−8.30	2.44
G6	26.8 ^c^	0.977	−12.98	−8.61	1.85
G7	27.1 ^c^	0.963	−11.54	−9.09	0.91
G2	15.1 ^d^	0.973	ND	ND	ND
MdxE (blank)	13.92 ^d^	0.952	-	-	-

Different letters indicate significant differences between ligands (Tukey’s test (*p* < 0.05); *p*-values of pairwise comparisons are shown in Table S1). ND = not detected.

## Data Availability

Data generated in this study are available from the authors upon reasonable request.

## Supplementary Materials

The following supporting information can be downloaded at: https://www.mdpi.com/article/10.3390/molecules28166080/s1, Figure S1: Gene clusters for alternative carbohydrate internalization pathways involving an ABC transporter and an extracellular glycosyl hydrolase (GH) from *T. mathranii* subsp. *mathranii*; Figure S2: MdxE-ANS binding optimization for H_o_ determination; Figure S3: Docked structures of the ANS fluorescence probe on the MdxE surface in open and closed conformations; Figure S4: Docked structures of G3–G7 in the sugar-binding site of MdxE; Table S1: Results of Tukey’s test for multiple comparisons of H_0_ from different CDs and linear dextrins; Table S2: Summary of docking simulations from MdxE in open and closed conformations with different CDs and linear dextrins; Table S3: Template information and statistical validation for homology models from elements involved in CD synthesis and cellular internalization.

## Sample Availability

Samples used in this study are available from the authors upon reasonable request.

## References

[1] Fiedler G., Pajatsch M., Böck A. (1996). Genetics of a Novel Starch Utilisation Pathway Present in *Klebsiella oxytoca*. J. Mol. Biol..

[2] Shim J.-H., Park J.-T., Hong J.-S., Kim K.W., Kim M.-J., Auh J.-H., Kim Y.-W., Park C.-S., Boos W., Kim J.-W. (2009). Role of Maltogenic Amylase and Pullulanase in Maltodextrin and Glycogen Metabolism of *Bacillus subtilis* 168. J. Bacteriol..

[3] Oslowski D.M., Jung J.-H., Seo D.-H., Park C.-S., Holden J.F. (2011). Production of Hydrogen from α-1,4- and β-1,4-Linked Saccharides by Marine Hyperthermophilic Archaea. Appl. Environ. Microbiol..

[4] Grégorio C. (2014). A Review: A History of Cyclodextrins. Chem. Rev..

[5] Uitdehaag J.C., van der Veen B.A., Dijkhuizen L., Dijkstra B.W. (2002). Catalytic mechanism and product specificity of cyclodextrin glycosyltransferase, a prototypical transglycosylase from the α-amylase family. Enzym. Microb. Technol..

[6] Janeček Š., Gabriško M. (2016). Remarkable evolutionary relatedness among the enzymes and proteins from the α-amylase family. Cell. Mol. Life Sci..

[7] Van der Veen B.A., van Alebeek G.W.M., Uitdehaag J.C.M., Dijkstra B.W., Dijkhuizen L. (2000). The three transglycosylation reactions catalyzed by cyclodextrin glycosyltransferase from Bacillus circulans (strain 251) proceed via different kinetic mechanisms. JBIC J. Biol. Inorg. Chem..

[8] Thomas A., Moinuddin K., Tretsiakova-McNally S., Joseph P. (2020). A Kinetic Analysis of the Thermal Degradation Behaviours of Some Bio-Based Substrates. Polymers.

[9] Poulson B.G., Alsulami Q.A., Sharfalddin A., El Agammy E.F., Mouffouk F., Emwas A.-H., Jaremko L., Jaremko M. (2021). Cyclodextrins: Structural, Chemical, and Physical Properties, and Applications. Polysaccharides.

[10] Pandolfo E., Caracciolo A.B., Rolando L. (2023). Recent Advances in Bacterial Degradation of Hydrocarbons. Water.

[11] Mousset E., Oturan N., van Hullebusch E.D., Guibaud G., Esposito G., Oturan M.A. (2014). Influence of solubilizing agents (cyclodextrin or surfactant) on phenanthrene degradation by electro-Fenton process—Study of soil washing recycling possibilities and environmental impact. Water Res..

[12] Shishido T.K., Jokela J., Kolehmainen C.-T., Fewer D.P., Wahlsten M., Wang H., Rouhiainen L., Rizzi E., De Bellis G., Permi P. (2015). Antifungal activity improved by coproduction of cyclodextrins and anabaenolysins in Cyanobacteria. Proc. Natl. Acad. Sci. USA.

[13] Centeno-Leija S., Espinosa-Barrera L., Velazquez-Cruz B., Cárdenas-Conejo Y., Virgen-Ortíz R., Valencia-Cruz G., Saenz R.A., Marín-Tovar Y., Gómez-Manzo S., Hernández-Ochoa B. (2022). Mining for novel cyclomaltodextrin glucanotransferases unravels the carbohydrate metabolism pathway via cyclodextrins in Thermoanaerobacterales. Sci. Rep..

[14] Pajatsch M., Gerhart M., Peist R., Horlacher R., Boos W., Böck A. (1998). The Periplasmic Cyclodextrin Binding Protein CymE from *Klebsiella oxytoca* and Its Role in Maltodextrin and Cyclodextrin Transport. J. Bacteriol..

[15] Hashimoto Y., Yamamoto T., Fujiwara S., Takagi M., Imanaka T. (2001). Extracellular Synthesis, Specific Recognition, and Intracellular Degradation of Cyclomaltodextrins by the Hyperthermophilic Archaeon *Thermococcus* sp. Strain B1001. J. Bacteriol..

[16] Thomas C., Tampé R. (2020). Structural and Mechanistic Principles of ABC Transporters. Annu. Rev. Biochem..

[17] Mächtel R., Narducci A., Griffith D.A., Cordes T., Orelle C. (2019). An integrated transport mechanism of the maltose ABC importer. Res. Microbiol..

[18] Leisico F., Godinho L.M., Gonçalves I.C., Silva S.P., Carneiro B., Romão M.J., Santos-Silva T., de Sá-Nogueira I. (2020). Multitask ATPases (NBDs) of bacterial ABC importers type I and their interspecies exchangeability. Sci. Rep..

[19] Van den Berg B., Prathyusha Bhamidimarri S., Dahyabhai Prajapati J., Kleinekathöfer U., Winterhalter M. (2015). Outer-membrane translocation of bulky small molecules by passive diffusion. Proc. Natl. Acad. Sci. USA.

[20] Kamionka A., Dahl M.K. (2001). *Bacillus subtilis* contains a cyclodextrin-binding protein which is part of a putative ABC-transporter. FEMS Microbiol. Lett..

[21] Tonozuka T., Sogawa A., Yamada M., Matsumoto N., Yoshida H., Kamitori S., Ichikawa K., Mizuno M., Nishikawa A., Sakano Y. (2007). Structural basis for cyclodextrin recognition by Thermoactinomyces vulgaris cyclo/maltodextrin-binding protein. FEBS J..

[22] Hülsmann A., Lurz R., Scheffel F., Schneider E. (2000). Maltose and Maltodextrin Transport in the Thermoacidophilic Gram-Positive Bacterium *Alicyclobacillus acidocaldarius* Is Mediated by a High-Affinity Transport System That Includes a Maltose Binding Protein Tolerant to Low pH. J. Bacteriol..

[23] Liu F., Liang J., Zhang B., Gao Y., Yang X., Hu T., Yang H., Xu W., Guddat L.W., Rao Z. (2020). Structural basis of trehalose recycling by the ABC transporter LpqY-SugABC. Sci. Adv..

[24] Hall J.A., Ganesan A.K., Chen J., Nikaido H. (1997). Two modes of ligand binding in maltose-binding protein of Escherichia coli: Functional significance in active transport. J. Biol. Chem..

[25] Medintz I.L., Deschamps J.R. (2006). Maltose-binding protein: A versatile platform for prototyping biosensing. Curr. Opin. Biotechnol..

[26] Gouridis G., Schuurman-Wolters G.K., Ploetz E., Husada F., Vietrov R., de Boer M., Cordes T., Poolman B. (2014). Conformational dynamics in substrate-binding domains influences transport in the ABC importer GlnPQ. Nat. Struct. Mol. Biol..

[27] De Boer M., Gouridis G., Vietrov R., Begg S.L., Schuurman-Wolters G.K., Husada F., Eleftheriadis N., Poolman B., McDevitt C.A., Cordes T. (2019). Conformational and dynamic plasticity in substrate-binding proteins underlies selective transport in ABC importers. Elife.

[28] Podkovyrov S.M., Zeikus J.G. (1992). Structure of the gene encoding cyclomaltodextrinase from Clostridium thermohydrosulfuricum 39E and characterization of the enzyme purified from *Escherichia coli*. J. Bacteriol..

[29] Zheng Y., Xue Y., Zhang Y., Zhou C., Schwaneberg U., Ma Y. (2010). Cloning, expression, and characterization of a thermostable glucoamylase from Thermoanaerobacter tengcongensis MB4. Appl. Microbiol. Biotechnol..

[30] Chen S., Liu J., Pei H., Li J., Zhou J., Xiang H. (2007). Molecular investigation of a novel thermostable glucan phosphorylase from Thermoanaerobacter tengcongensis. Enzym. Microb. Technol..

[31] Li Z., Jiang N., Yang K., Zheng J. (2016). Cloning, expression, and characterization of a thermostable glucose-6-phosphate dehydrogenase from Thermoanaerobacter tengcongensis. Extremophiles.

[32] Lee H.-S., Shockley K.R., Schut G.J., Conners S.B., Montero C.I., Johnson M.R., Chou C.-J., Bridger S.L., Wigner N., Brehm S.D. (2006). Transcriptional and Biochemical Analysis of Starch Metabolism in the Hyperthermophilic Archaeon *Pyrococcus furiosus*. J. Bacteriol..

[33] Labes A., Schönheit P. (2007). Unusual Starch Degradation Pathway via Cyclodextrins in the Hyperthermophilic Sulfate-Reducing Archaeon *Archaeoglobus fulgidus* Strain 7324. J. Bacteriol..

[34] Davidson A.L., Chen J. (2004). ATP-Binding Cassette Transporters in Bacteria. Annu. Rev. Biochem..

[35] Schäfer K., Magnusson U., Scheffel F., Schiefner A., Sandgren M.O., Diederichs K., Welte W., Hülsmann A., Schneider E., Mowbray S.L. (2004). X-ray Structures of the Maltose–Maltodextrin-binding Protein of the Thermoacidophilic Bacterium Alicyclobacillus acidocaldarius Provide Insight into Acid Stability of Proteins. J. Mol. Biol..

[36] Matsumoto N., Yamada M., Kurakata Y., Yoshida H., Kamitori S., Nishikawa A., Tonozuka T. (2009). Crystal structures of open and closed forms of cyclo/maltodextrin-binding protein. FEBS J..

[37] Duan X., Hall J.A., Nikaido H., Quiocho F.A. (2001). Crystal structures of the maltodextrin/maltose-binding protein complexed with reduced oligosaccharides: Flexibility of tertiary structure and ligand binding. J. Mol. Biol..

[38] Spiwok V. (2017). CH/π Interactions in Carbohydrate Recognition. Molecules.

[39] Quiocho F.A., Spurlino J.C., Rodseth L.E. (1997). Extensive features of tight oligosaccharide binding revealed in high-resolution structures of the maltodextrin transport/chemosensory receptor. Structure.

[40] van der Veen B.A., Uitdehaag J.C.M., Dijkstra B.W., Dijkhuizen L. (2000). The role of arginine 47 in the cyclization and coupling reactions of cyclodextrin glycosyltransferase from Bacillus circulans strain 251. JBIC J. Biol. Inorg. Chem..

[41] Ohtaki A., Kondo S., Shimura Y., Tonozuka T., Sakano Y., Kamitori S. (2001). Role of Phe286 in the recognition mechanism of cyclomaltooligosaccharides (cyclodextrins) by Thermoactinomyces vulgaris R-47 α-amylase 2 (TVAII). X-ray structures of the mutant TVAIIs, F286A and F286Y, and kinetic analyses of the Phe286-replaced mutant TVAIIs. Carbohydr. Res..

[42] Jones C.R., Ray M., Dawson K.A., Strobel H.J. (2000). High-Affinity Maltose Binding and Transport by the Thermophilic Anaerobe *Thermoanaerobacter ethanolicus* 39E. Appl. Environ. Microbiol..

[43] Labes A., Schönheit P. (2001). Sugar utilization in the hyperthermophilic, sulfate-reducing archaeon Archaeoglobus fulgidus strain 7324: Starch degradation to acetate and CO2 via a modified Embden-Meyerhof pathway and acetyl-CoA synthetase (ADP-forming). Arch. Microbiol..

[44] Schönert S., Seitz S., Krafft H., Feuerbaum E.-A., Andernach I., Witz G., Dahl M.K. (2006). Maltose and Maltodextrin Utilization by *Bacillus subtilis*. J. Bacteriol..

[45] Shukla S., Bafna K., Gullett C., Myles D.A.A., Agarwal P.K., Cuneo M.J. (2018). Differential Substrate Recognition by Maltose Binding Proteins Influenced by Structure and Dynamics. Biochemistry.

[46] Lu C., Quiocho G.Y.A., Sharff F.A., Rodseth A.J., Spurlino L.E., Quiocho J.C. (1993). Refined 1.8-A Structure Reveals the Mode of Binding of P-Cyclodextrin to the Maltodextrin Binding Protein. Biochemistry.

[47] De la Cruz-Torres L.F., Rodríguez-Celestino V., Centeno-Leija S., Serrano-Posada H., Ceballos-Magaña S.G., Aguilar-Padilla J., Mancilla-Margalli N.A., Osuna-Castro J.A. (2022). Development of a rapid, high-sensitivity, low-cost fluorescence method for protein surface hydrophobicity determination using a Nanodrop fluorospectrometer. Food Chem..

[48] Deshpande M., Sathe S.K. (2018). Interactions with 8-Anilinonaphthalene-1-sulfonic Acid (ANS) and Surface Hydrophobicity of Black Gram (*Vigna mungo*) Phaseolin. J. Food Sci..

[49] Thompson J.D., Higgins D.G., Gibson T.J. (1994). CLUSTAL W: Improving the sensitivity of progressive multiple sequence alignment through sequence weighting, position-specific gap penalties and weight matrix choice. Nucleic Acids Res..

[50] Oldham M.L., Chen J. (2011). Snapshots of the maltose transporter during ATP hydrolysis. Proc. Natl. Acad. Sci. USA.

[51] Oldham M.L., Chen S., Chen J. (2013). Structural basis for substrate specificity in the *Escherichia coli* maltose transport system. Proc. Natl. Acad. Sci. USA.

[52] Oldham M.L., Chen J. (2011). Crystal Structure of the Maltose Transporter in a Pretranslocation Intermediate State. Science.

[53] Wen P.-C., Tajkhorshid E. (2011). Conformational Coupling of the Nucleotide-Binding and the Transmembrane Domains in ABC Transporters. Biophys. J..

[54] Chen J., Lu G., Lin J., Davidson A.L., Quiocho F.A. (2003). A Tweezers-like Motion of the ATP-Binding Cassette Dimer in an ABC Transport Cycle. Mol. Cell.

[55] Khare D., Oldham M.L., Orelle C., Davidson A.L., Chen J. (2009). Alternating Access in Maltose Transporter Mediated by Rigid-Body Rotations. Mol. Cell.

[56] Bradford M.M. (1976). A rapid and sensitive method for the quantitation of microgram quantities of protein utilizing the principle of protein-dye binding. Anal. Biochem..

[57] Aguilar-Padilla J., Centeno-Leija S., Bojórquez-Velázquez E., Elizalde-Contreras J.M., Ruiz-May E., Serrano-Posada H., Osuna-Castro J.A. (2023). Characterization of the Technofunctional Properties and Three-Dimensional Structure Prediction of 11S Globulins from Amaranth (*Amaranthus hypochondriacus* L.) Seeds. Foods.

[58] Lenth R.V., Bolker B., Buerkner P., Giné-Vázquez I., Herve M., Jung M., Love J., Miguez F., Riebl H., Singmann H. (2023). emmeans: Estimated Marginal Means, Aka Least-Squares Means. R Package Version 1.8.7. https://CRAN.R-project.org/package=emmeans.

[59] R core Team (2023). A Language and Environment for Statistical Computing. R Foundation for Statistical Computing. https://www.r-project.org/.

[60] Waterhouse A., Bertoni M., Bienert S., Studer G., Tauriello G., Gumienny R., Heer F.T., De Beer T.A.P., Rempfer C., Bordoli L. (2018). SWISS-MODEL: Homology modelling of protein structures and complexes. Nucleic Acids Res..

[61] Emsley P., Lohkamp B., Scott W.G., Cowtan K. (2010). Features and development of Coot. Acta Crystallogr. D Biol. Crystallogr..

[62] Land H., Humble M.S. (2018). YASARA: A tool to obtain structural guidance in biocatalytic investigations. Methods Mol. Biol..

[63] Williams C.J., Headd J.J., Moriarty N.W., Prisant M.G., Videau L.L., Deis L.N., Verma V., Keedy D.A., Hintze B.J., Chen V.B. (2018). MolProbity: More and better reference data for improved all-atom structure validation. Protein Sci..

[64] Morris G.M., Huey R., Lindstrom W., Sanner M.F., Belew R.K., Goodsell D.S., Olson A.J. (2009). AutoDock4 and AutoDockTools4: Automated docking with selective receptor flexibility. J. Comput. Chem..

[65] Kim S., Chen J., Cheng T., Gindulyte A., He J., He S., Li Q., Shoemaker B.A., Thiessen P.A., Yu B. (2023). PubChem 2023 update. Nucleic Acids Res..

[66] Hanwell M.D., Curtis D.E., Lonie D.C., Vandermeersch T., Zurek E., Hutchison G.R. (2012). SOFTWARE Open Access Avogadro: An advanced semantic chemical editor, visualization, and analysis platform. J. Cheminformatics.

[67] (2021). BIOVIA DS Discovery Studio Modeling Enviroment. Dassault System. https://www.3ds.com/products-services/biovia/products/molecular-modeling-simulation/biovia-discovery-studio/.

[68] Pettersen E.F., Goddard T.D., Huang C.C., Meng E.C., Couch G.S., Croll T.I., Morris J.H., Ferrin T.E. (2021). UCSF ChimeraX: Structure visualization for researchers, educators, and developers. Protein Sci..

