# Supplementary material for: Binding Specificity of a Novel Cyclo/Maltodextrin-Binding Protein and Its Role in the Cyclodextrin ABC Importer System from Thermoanaerobacterales
Source: Molecules. 2023 Aug 16;28(16):6080. doi: 10.3390/molecules28166080 (PMC10458862; doi:10.3390/molecules28166080)
Supplement: Supplementary file 1 [file molecules-28-06080-s001.zip › molecules-2547693-supplementary.pdf]

Supporting information to:

Binding specificity of a novel cyclo/maltodextrin-binding protein and its role in the cyclodextrin ABC importer system from Thermoanaerobacterales

Jorge Aranda-Caraballo, Roberto A. Saenz, Alonso A. López-Zavala, Beatriz Velazquez-Cruz, Laura Espinosa-Barrera, Yair Cárdenas-Conejo, Andrés Zárate-Romero, Oscar Linares-Vergara, Juan A. Osuna-Castro, Edgar Bonales-Alatorre, Sara Centeno-Leija, and Hugo Serrano-Posada

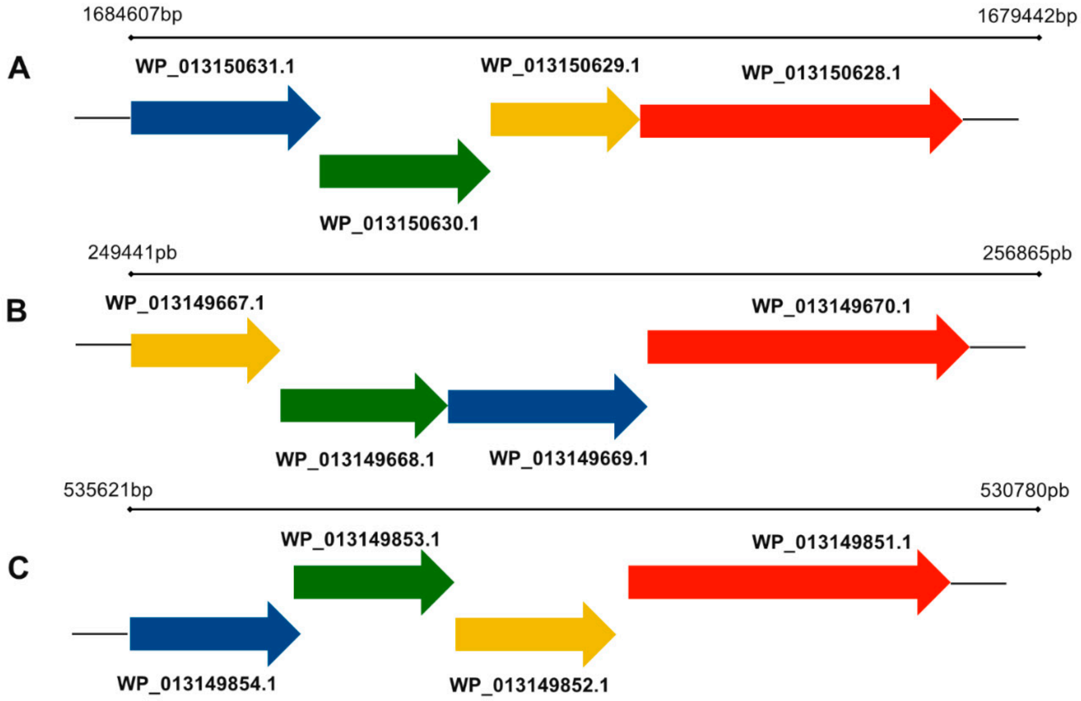

Figure S1. Gene clusters for alternative carbohydrate internalization pathways involving an ABC transporter and an extracellular glycosyl hydrolase (GH) from *T. mathranii* subsp. *mathranii* (NCBI Taxonomy ID: 583358). Note the GH-encoding gene (red arrow) involved in each pathway: **(A)** alpha-mannosidase, **(B)** beta-galactosidase, and **(C)** maltogenic α-amylase. Proteins from the ABC transporters are also indicated in different colors: SBPs (blue arrow), permeases F (green arrow), and G (yellow arrow).

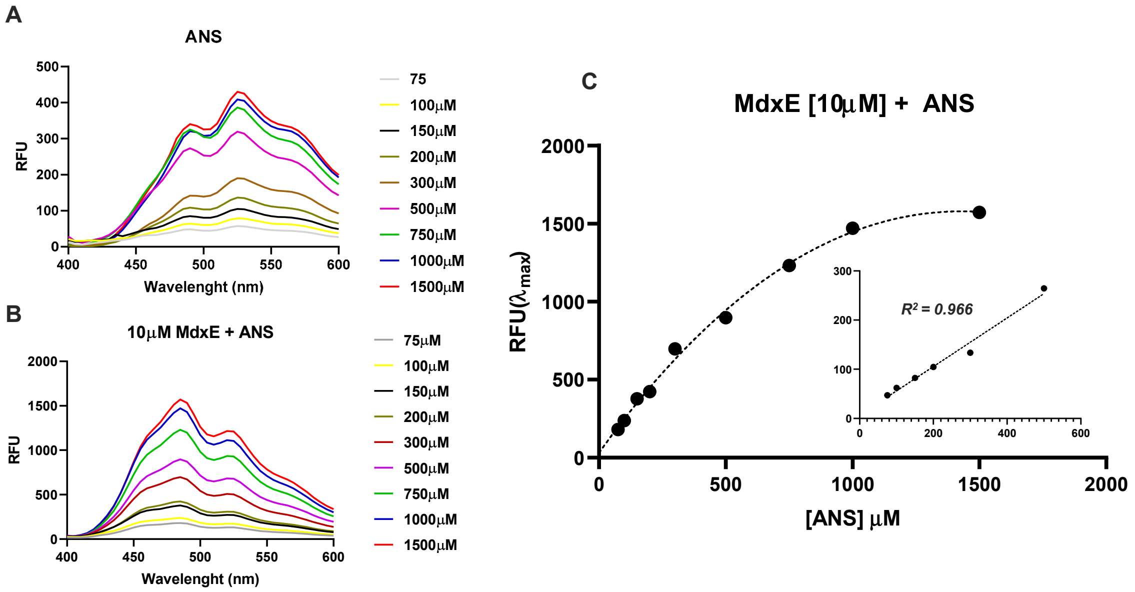

Figure S2. MdxE-ANS binding optimization for H_o determination. **(A)** Increment of intensity (RFU) as a function of ANS concentration. **(B)** The binding capacity of ANS to MdxE (10 µM) by fluorescence measurements. **(C)** Linearity range for the increase in fluorescent response at λ_{max}= 485 nm as a function of ANS concentration. Note that the scans were taken in the 400-600 nm range.

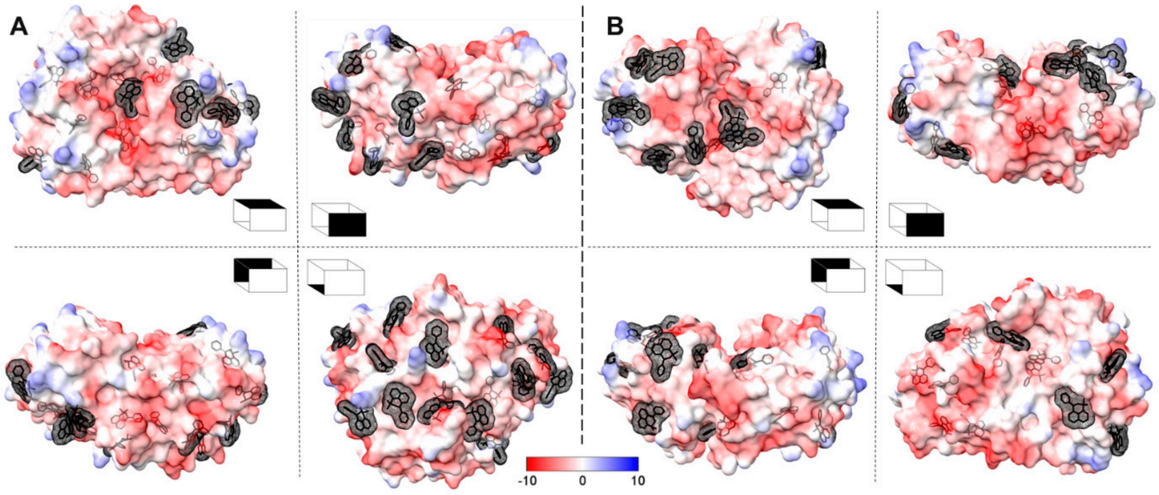

Figure S3. Docked structures of the ANS fluorescence probe on the MdxE surface in open and closed conformations. The surface electrostatic potential map of MdxE in closed **(A)** and open **(B)** conformations shows the potential binding regions for ANS (black meshed surface). The negatively and positively charged surfaces of MdxE are colored red and blue, respectively, and the hydrophobic surface is colored white. The electrostatic surface was calculated in ChimeraX using the default coulombic values.

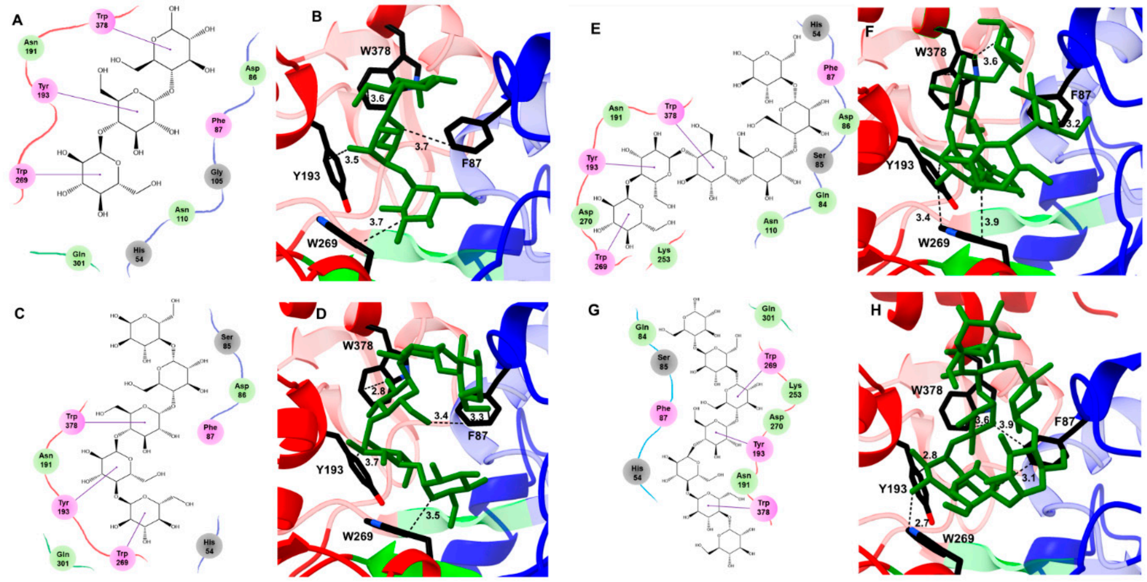

Figure S4. Docked structures of G3-G7 in the sugar-binding site of MdxE. **(A,B)** MdxE/G3. **(C,D)** MdxE/G5. **(E,F)** MdxE/G6. **(G,H)** MdxE/G7. The two-dimensional (2D) interaction plots show the hydrogen bonds between glucose (G1) and the side chain of a residue in green, hydrophobic interactions in violet, and hydrogen bonds with the main chain atoms in gray. The 3D docked structures exhibit the key residues (black cylinders) in the N-domain (blue) and C-domain (red) involved in ligand recognition. The residues in the 2D interaction plots are linked with red, blue, and green lines representing the C-domain, N-domain, and hinge regions, respectively. Note that the absence of Glu155 from the hinge-I region in 2D interaction plots for all complexes might affect obtaining the MdxE closed form. Distances are in Å

Table S1. Results of Tukey's test for multiple comparisons of H_0 from different CDs and linear dextrins.

Contrast	Estimate	SE	df	t.ratio	p.value
**blank – α-CD**	-23.25	1.22	171	-19.02	0
**blank – β-CD**	-27.95	1.22	171	-22.86	0
**blank – γ-CD**	-10.58	1.22	171	-8.658	2.00E-13
**blank - G2**	-1.194	1.22	171	-0.9767	0.9875
**blank - G3**	-10.44	1.22	171	-8.544	3.22E-13
**blank - G5**	-13.45	1.22	171	-11.00	3.43E-14
**blank - G6**	-12.97	1.22	171	-10.61	5.72E-14
**blank - G7**	-13.14	1.22	171	-10.74	4.90E-14
**α-CD - β-CD**	-4.701	1.22	171	-3.846	0.005198
**α-CD - γ-CD**	12.66	1.22	171	10.36	6.43E-14
**α-CD - G2**	22.05	1.22	171	18.04	0
**α-CD - G3**	12.80	1.22	171	10.47	6.01E-14
**α-CD - G5**	9.801	1.22	171	8.017	5.91E-12
**α-CD - G6**	10.27	1.22	171	8.408	6.36E-13
**α-CD - G7**	10.11	1.22	171	8.270	1.37E-12
**β-CD - γ-CD**	17.36	1.22	171	14.20	0
**β-CD - G2**	26.76	1.22	171	21.88	0
**β-CD - G3**	17.50	1.22	171	14.32	0
**β-CD - G5**	14.50	1.22	171	11.86	0
**β-CD - G6**	14.98	1.22	171	12.25	0
**β-CD - G7**	14.81	1.22	171	12.11	0
**γ-CD - G2**	9.391	1.22	171	7.681	4.19E-11
**γ-CD - G3**	0.1392	1.22	171	0.1139	1
**γ-CD - G5**	-2.866	1.22	171	-2.344	0.3219
**γ-CD - G6**	-2.388	1.22	171	-1.953	0.5778
**γ-CD - G7**	-2.556	1.22	171	-2.091	0.4827
**G2 - G3**	-9.251	1.22	171	-7.567	8.08E-11
**G2 - G5**	-12.25	1.22	171	-10.02	7.09E-14
**G2 - G6**	-11.77	1.22	171	-9.635	7.23E-14
**G2 - G7**	-11.94	1.22	171	-9.772	7.32E-14
**G3 - G5**	-3.005	1.22	171	-2.458	0.2600
**G3 - G6**	-2.527	1.22	171	-2.067	0.4990
**G3 - G7**	-2.695	1.22	171	-2.205	0.4071
**G5 - G6**	0.4779	1.22	171	0.3909	0.9999
**G5 - G7**	0.3095	1.22	171	0.2531	0.9999
**G6 - G7**	-0.1684	1.22	171	-0.1377	1

Table S2. Summary of docking simulations from MdxE in open and closed conformations with different CDs and linear dextrins.

Ligand	Closed Form ΔG (kcal mol^{-1})	Open Form ΔG (kcal mol^{-1})
α-CD	-10.8	-8.6
β-CD	-9.7	-8.5
γ-CD	-10.8	-8.7
G3	-7.3	-6.7
G5	-7.4	-6.1
G6	-9.1	-4.2
G7	-4.5	-4.0
G2	-6.8	-5.2

Table S3. Template information and statistical validation for homology models from elements involved in CD synthesis and cellular internalization.

Protein	Template (PDB ID)	GMQE *	QMEANDisCo **	Resolution (Å)	Sequence identity (%)
ThmA	6WNI	0.95	0.94	1.6	83
MdxE (*closed*)	1URD	0.81	0.81	1.5	43
MdxE (*open*)	2ZYO	0.70	0.71	1.5	34
MdxF	3PUV	0.62	0.71	2.4	41
MdxG	3PUV	0.51	0.67	2.4	36
MsmX	1Q12	0.70	0.68	2.3	47

* GMQE: Global Model Quality Estimate.

** QMEANDisCo: Scoring function for global and local absolute quality on the model. Ensembles of distance constraints (DisCo) are used to assess the agreement of pairwise residue-residue distance.
